# Evolving principles underlying neural lineage conversion and their relevance for biomedical translation

**DOI:** 10.12688/f1000research.18926.1

**Published:** 2019-08-30

**Authors:** Lea Jessica Flitsch, Oliver Brüstle

**Affiliations:** 1 Institute of Reconstructive Neurobiology, University of Bonn School of Medicine & University Hospital Bonn, Bonn, North Rhine Wesphalia, 53127, Germany

**Keywords:** Cell programming, Direct conversion, Transdifferentiation, Forward programming, In vivo conversion, Translation, Disease modelling, Transplantation

## Abstract

Scientific and technological advances of the past decade have shed light on the mechanisms underlying cell fate acquisition, including its transcriptional and epigenetic regulation during embryonic development. This knowledge has enabled us to purposefully engineer cell fates *in vitro* by manipulating expression levels of lineage-instructing transcription factors. Here, we review the state of the art in the cell programming field with a focus on the derivation of neural cells. We reflect on what we know about the mechanisms underlying fate changes in general and on the degree of epigenetic remodeling conveyed by the distinct reprogramming and direct conversion strategies available. Moreover, we discuss the implications of residual epigenetic memory for biomedical applications such as disease modeling and neuroregeneration. Finally, we cover recent developments approaching cell fate conversion in the living brain and define questions which need to be addressed before cell programming can become an integral part of translational medicine.

## An introduction to cell programming and lineage conversion

The term cellular programming describes the modulation of transcriptional networks underlying cell identity. Research on cell programming has a remarkable history. First reports describing the principal feasibility of converting one cell type into another were published as early as 1987, when Davis, Weintraub, and Lassar derived myoblasts by overexpressing the myoblast transcription factor (TF) Myod3 in a mouse fibroblast line ^1^. However, at that time, cellular programming was restricted to the conversion of lineage-related cells of the same germ layer (see the 2009 review by Graf and Enver ^2^). This changed dramatically when Kazutoshi Takahashi and Shinya Yamanaka revealed that overexpression of the four TFs Oct3/4, Sox2, Klf4, and c-Myc is sufficient to induce a pluripotent state in mouse ^3^ and human ^4^ fibroblasts (for a more detailed review, see 5). Today, protocols are available to obtain these embryonic stem cell (ESC)-like induced pluripotent stem cells (iPSCs) from various species and starting cell types.

The success of the iPSC approach demonstrated that cell programming is not restricted to the conversion of related cell types and fueled attempts to achieve somatic-to-somatic cell conversion across germ layers. One avenue pursued in this direction has been the combination of time-restricted expression of the classic iPSC reprogramming TF cocktails with growth factors and small molecules promoting neural lineage development. An exemplar for such a “partial” reprogramming is the Oct3/4-, Sox2-, Klf4-, and c-Myc-driven derivation of neural stem cells (NSCs) from fibroblasts ^6– 10^ or blood cells ^11^, where transgene expression was combined with an exposure to FGF2, FGF4, and/or EGF ^6, 7, 10, 11^, FGF2 and/or EGF in conjunction with LIF ^8^, or LIF in combination with the TGFβ-inhibitor SB431542 and the GSK3β-inhibitor CHIR99021 ^9^. It is worth mentioning that in accordance with the transient expression of TFs used for generating iPSCs, partial reprogramming to NSCs may involve a short transit through a pluripotency-like state and can result in mixed cultures of iPSCs and NSCs ^12, 13^. Interestingly, such a pluripotency transit can also occur without forced Oct4 expression. Lineage tracing using an Oct4 reporter revealed that NSCs derived by overexpression of Sox2, Klf4, and c-Myc in conjunction with the neural-specific TF Brn4 instead of Oct4—a protocol originally published by Han *et al*. ^14^ in 2012—originate from Oct4-expressing iPSC-like cells ^12^. Such a pluripotency transit can be used to mechanistically discriminate partial reprogramming from direct cell fate conversion (hereafter also denoted as transdifferentiation) based on the overexpression of lineage-specific TFs.

A major breakthrough concerning transdifferentiation across germ layers was in 2010, when the group of Marius Wernig succeeded in inducing neurons from mouse fibroblasts by overexpressing the neural lineage-specific TFs Ascl1, Brn2, and Myt1l ^15^. Soon thereafter, conversion of human fibroblasts to induced neurons (iNs) was achieved by using exactly this ASCL1, BRN2, and MYT1L TF combination ^16, 17^; ASCL1, BRN2, and MYT1L in conjunction with NEUROD1 ^18^; or BRN2 and MYT1L together with the neuronal microRNA miR124 ^19^ ( Table 1).

**Table 1.** Transcription factor–based generation of induced neurons *in vitro*.

Derived cell type	Starting cell type	Species	Transcription factors used for reprogramming	Reference
Trans-germ layer conversion				
Neurons (generic)	Fibroblasts	Mouse	Ascl1, Brn2, Myt1l	Vierbuchen *et al*. (2010) ^15^
	Fibroblasts, Hepatocytes	Mouse	Ascl1, Brn2, Myt1l	Marro *et al*. (2011) ^44^
	Fibroblasts	Mouse	Ascl1, Brn2, Myt1l	Adler *et al*. (2012) ^45^
	Fibroblasts	Mouse	Ascl1, Brn2 and Myt1l or Ascl1, Brn2, Ngn2	Meng *et al*. (2012) ^46^
	Fibroblasts	Mouse	None (chemical reprogramming)	Li *et al*. (2015) ^47^
	Fibroblasts	Mouse	Several (CRISPR activation screen)	Liu *et al*. (2018) ^48^
	Fibroblasts	Mouse	Several (TF screen)	Tsunemoto *et al*. (2018) ^49^
	Fibroblasts ( *in situ*)	Mouse, Human	Ascl1, Brn2, Myt1l	Torper *et al*. (2013) ^50^
	Fibroblasts	Human	miR124, BRN2, MYT1L	Ambasudhan *et al*. (2011) ^19^
	Fibroblasts	Human	ASCL1, BRN2, MYT1L, NEUROD1	Pang *et al*. (2011) ^18^
	Fibroblasts	Human	ASCL1, BRN2, MYT1L	Pfisterer *et al*. (2011) ^17^
	Fibroblasts	Human	miR9/9* and miR124 (+ ASCL1, MYT1L and/or NEUROD2)	Yoo *et al*. (2011) ^51^
	Fibroblasts	Human	ASCL1, NGN2	Ladewig *et al*. (2012) ^52^
	Fibroblasts	Human	miR-124 regulated ASCL1, BRN2, MYT1L	Lau *et al*. (2014) ^53^
	Fibroblasts	Human	ASCL1, BRN2, MYT1L	Pereira *et al*. (2014) ^54^
	Fibroblasts	Human	shp16 and/or shp19 or hTERT	Sun *et al*. (2014) ^55^
	Fibroblasts	Human	ASCL1, NGN2 (Ladewig *et al*. (2012) ^52^)	Mertens *et al*. (2015) ^56^
	Fibroblasts	Human	miR9/9*, miR124 (Yoo *et al*. (2011) ^51^)	Huh *et al*. (2016) ^57^
	Fibroblasts	Human	ASCL1, BRN2, MYT1L (Pereira *et al*. (2014) ^54^)	Pfisterer *et al*. (2016) ^58^
	Fibroblasts	Human	NGN2	Smith *et al*. (2016) ^59^
	Fibroblasts	Human	ASCL1, BRN2 (+ shRNA REST)	Drouin-Ouellet *et al*. (2017) ^60^
	Fibroblasts	Human	ASCL1, NGN2 (Mertens *et al*. (2015) ^56^)	Kim *et al*. (2018) ^61^
	Fibroblasts	Human	ASCL1, NGN2	Herdy *et al*. (2019) ^62^
	Microglia	Mouse	Neurod1	Matsuda *et al*. (2019) ^35^
Glutamatergic neurons	Fibroblasts	Mouse, Human	Ascl1	Chanda *et al*. (2014) ^63^
	Fibroblasts	Human	None (chemical reprogramming)	Hu *et al*. (2015) ^64^
	Fibroblasts	Human	BRN2, MYT1L, FEZF2	Miskinyte *et al*. (2017) ^65^
GABAergic neurons	Adipose-derived stem cells	Human	None (chemical reprogramming)	Park *et al*. (2017) ^66^
	Fibroblasts	Mouse	Ascl1	Shi *et al*. (2016) ^67^
	Pericytes	Human	ASCL1, SOX2	Karow *et al*. (2012) ^33^
	Pericytes	Human	ASCL1, SOX2 (Karow *et al*. (2012) ^33^)	Karow *et al*. (2018) ^34^
Midbrain dopamine-like neurons	Fibroblasts	Mouse	Ascl1, Nurr1, Lmx1a, Pitx3, Foxa2, En1	Kim *et al*. (2011) ^68^
	Fibroblasts	Mouse, Human	Ascl1, Nurr1, Lmx1a	Caiazzo *et al*. (2011) ^69^
	Fibroblasts ( *in situ*)	Mouse, Human	Ascl1, Brn2, Myt1l, Lmx1a, Lmx1b, Foxa2, Otx2	Torper *et al*. (2013) ^50^
	Fibroblasts	Human	ASCL1, BRN2, MYT1L, LMX1A, FOXA2	Pfisterer *et al*. (2011) ^16^
	Fibroblasts	Human	ASCL1, BRN2, MYT1L, LMX1A, LMX1B, FOXA2, OTX2	Pereira *et al*. (2014) ^54^
	Fibroblasts	Human	ASCL1, NURR1, LMX1A, miR124 (+shp53)	Jiang *et al*. (2015) ^70^
Striatal medium spiny neurons	Fibroblasts	Human	miR9/9*, miR124, CTIP2, DLX1, DLX2, MYT1L	Victor *et al*. (2014) ^71^
	Fibroblasts	Human	miR9/9*, miR124, CTIP2, DLX1, DLX2, MYT1L (Victor *et al*. (2014) ^71^)	Victor *et al*. (2018) ^72^
Serotonergic neurons	Fibroblasts	Human	ASCL1, NGN2, NKX2.2, FEV, GATA2, LMX1B	Vadodaria *et al*. (2016) ^73^
	Fibroblasts	Human	ASCL1, FEV, LMX1B, FOXA2 (+ shp53)	Xu *et al*. (2016) ^74^
Motoneurons	Fibroblasts	Mouse	Ascl1, Brn2, Myt1l, Ngn2, Lhx3, Hb9, Isl1	Ichida *et al*. (2018) ^75^
	Fibroblasts	Mouse, Human	Ascl1, Brn2, Myt1l, Ngn2, Lhx3, Hb9, Sox1, Pax6, Nkx6.1, Olig2 (+ Isl1)	Son *et al*. (2011) ^76^
	Fibroblasts	Human	NGN2, SOX11, ISL1, LHX3	Liu *et al*. (2016) ^77^
	Fibroblasts	Human	miR9/9*, miR124, ISL1, LHX3	Abernathy *et al*. (2017) ^78^
	Fibroblasts	Human	NGN2, SOX11, ISL1, LHX3	Tang *et al*. (2017) ^79^
Sensory neurons	Fibroblasts	Mouse, Human	Brn3a, Ngn1 or Brn3a, Ngn2	Blanchard *et al*. (2015) ^80^
	Fibroblasts	Mouse, Human	Ascl1, Myt1l, Ngn1, Isl2, Klf7	Wainger *et al*. (2015) ^81^
Intra-germ layer conversion				
Neurons	Astrocytes	Mouse	Ngn2 or Ascl1	Berninger *et al*. (2007) ^40^
	Astrocytes	Mouse	Ngn2 or Ascl1 or Dlx2 (+ Ascl1)	Heinrich *et al*. (2010) ^82^
	Astrocytes	Mouse	Ascl1 (+ Bcl2)	Gascón *et al*. (2016) ^83^
	Astrocytes ( *in situ*)	Mouse, Human	Ascl1, Brn2, Myt1l	Torper *et al*. (2013) ^50^
	Astrocytes	Human	OCT4, SOX2, or NANOG	Corti *et al*. (2012) ^41^
	Astrocytes	Human	miR302/367	Ghasemi-Kasman *et al*. (2015) ^42^
	Astrocytes	Human	None (chemical reprogramming)	Zhang *et al*. (2015) ^43^
Midbrain dopamine-like neurons	Astrocytes ( *in situ*)	Mouse, Human	Ascl1, Brn2, Myt1l, Lmx1a, Lmx1b, Foxa2, Otx2	Torper *et al*. (2013) ^50^
	Astrocytes	Human	ASCL1, NEUROD1, LMX1A, miR218	Rivetti di Val Cervo *et al*. (2017) ^84^

While direct transdifferentiation into a neuron remains a fascinating concept, the applicability of this approach can be limited by the fact that neurons are post-mitotic, thereby restricting large-scale applications. In addition, since not all cells undergo successful transdifferentiation, elimination of partially reprogrammed cells remains an issue. Finally, each transdifferentiated neuron represents a singular event and thus cannot be subjected to common batch control-based quality-control regimens, limiting the degree of standardization that can be reached with iN cultures. In light of this, expandable NSCs or neural progenitor cells (NPCs) could offer an interesting alternative. Indeed, several groups reported on the successful transdifferentiation of mouse ^20, 21^ and human ^22– 27^ fibroblasts into still-proliferative NSCs or NPCs using different NSC-enriched TFs or TF combinations ( Table 2). Subsequently, other somatic cells, too, were found to be amenable to direct neural conversion. In this context, easily accessible cell populations such as blood-derived ^28– 31^ and urine-derived ^32^ cells are of particular interest. Alongside converting non-central nervous system (non-CNS)-resident cells, there has been significant progress with neural conversion of non-neural, CNS-resident cells such as brain pericytes ^33, 34^ and yolk sac-born microglia ^35^, which both represent attractive candidates for *in vivo* reprogramming. In parallel, transdifferentiation of astrocytes—which can be regarded as derivatives of neurogenic radial glia cells at the end of neural development (for further details on the relationship of radial glia cells, NSCs, and neurogenesis, see Falk and Götz ^36^)—has been rapidly developing ^37– 39^, although most studies have been focusing on transdifferentiating astrocytes *in vivo* (see “Destabilizing and converting cell fates *in vivo*” section below and Table 3). As for the *in vitro* conversion of astrocytes into neurons, Benedikt Berninger, Magdalena Götz, and colleagues already showed in 2007 that this can be achieved by overexpression of the single neurogenic TF Ngn2 or Ascl1 ^40^. In the meantime, additional paradigms based on TF combinations ^41^, microRNAs ^42^, or small molecules ^43^ (or a combination of these) have been reported for astrocyte-to-neuron conversion.

**Table 2.** Approaches for the direct *in vitro* conversion of somatic cells into neural stem cells/neural progenitor cells.

Starting cell type	Species	Transcription factors used for reprogramming	Reference
Trans-germ layer conversion			
Cord blood cells (CD133 ^+^)	Human	SOX2, c-MYC	Giorgetti *et al*. (2012) ^28^
Cord blood cells (CD133 ^+^)	Human	SOX2, c-Myc	Castano *et al*. (2016) ^29^
Cord blood cells (CD34 ^+^)	Human	OCT4	Liao *et al*. (2015) ^85^
Cord blood cells (CD34 ^+^), Fibroblasts	Human	SOX2, HMGA2	Yu *et al*. (2015) ^24^
Cord blood cells (CD34 ^+^), Peripheral blood cells	Human	SOX2, c-MYC	Sheng *et al*. (2018) ^30^
Peripheral blood cells (CD34 ^+^)	Human	OCT3/4, SOX2, KLF4, c-MYC	Wang *et al*. (2013) ^11^
Peripheral blood cells	Human	OCT4, SOX2, KLF4, c-MYC, LIN28, NANOG, SV40LT	Tang *et al*. (2016) ^86^
Peripheral blood cells, Fibroblasts	Human	SOX2, KLF4, BRN2, ZIC3	Thier *et al*. (2019) ^31^
Fibroblasts	Mouse	Oct4, Sox2, Klf4, c-Myc	Kim *et al*. (2011) ^6^
Fibroblasts	Mouse	Brn4, Sox2, Klf4, c-Myc (+ Tcf3)	Han *et al*. (2012) ^14^
Fibroblasts	Mouse	Brn2, Sox2, Foxg1	Lujan *et al*. (2012) ^20^
Fibroblasts	Mouse	Oct4, Sox2, Klf4, c-Myc	Matsui *et al*. (2012) ^8^
Fibroblasts	Mouse	None (chemical reprogramming)	Cheng *et al*. (2014) ^87^
Fibroblasts	Mouse	Oct4, Sox2, Klf4, c-Myc	Thier *et al*. (2012) ^7^
Fibroblasts	Mouse	Sox2, c-Myc, Brn2, Nr2e, Bmi1	Tian *et al*. (2012) ^21^
Fibroblasts	Mouse	None (chemical reprogramming)	Han *et al*. (2016) ^88^
Fibroblasts	Mouse	Brn4, Sox2, Klf4, c-Myc	Kim *et al*. (2016) ^89^
Fibroblasts	Mouse	None (chemical reprogramming)	Zhang *et al*. (2016) ^90^
Fibroblasts	Mouse	None (chemical reprogramming)	Zheng *et al*. (2016) ^91^
Fibroblasts	Mouse, Human	Sox2	Ring *et al*. (2012) ^22^
Fibroblasts	Mouse, Human	Ptf1a	Xiao *et al*. (2018) ^27^
Fibroblasts	Pig	Oct4, Sox2, Klf4, l-Myc, Lin28	Xu *et al*. (2014) ^92^
Fibroblasts	Monkey, Human	Oct4, Sox2, Klf4, c-Myc	Lu *et al*. (2013) ^9^
Fibroblasts	Human	SOX2, PAX6	Maucksch *et al*. (2012) ^93^
Fibroblasts	Human	OCT4, SOX2, KLF4, c-MYC	Meyer *et al*. (2014) ^10^
Fibroblasts	Human	Oct4	Zhu *et al*. (2014) ^94^
Fibroblasts	Human	SOX2	Mirakhori *et al*. (2015) ^95^
Fibroblasts	Human	OCT3/4, SOX2, KLF4, l-Myc, LIN28, shp53	Capetian *et al*. (2016) ^96^
Fibroblasts	Human	ZFP521	Shabazi *et al*. (2016) ^25^
Fibroblasts	Human	CBX2, HES1, ID1, TFAP2A, ZFP42, ZNF423 or FOXG1, GATA3, NR2A2, PAX6, SALL2, TFAP2A, ZFP42	Hou *et al*. (2017) ^26^
Fibroblasts	Human	SOX2, PAX6	Connor *et al*. (2018) ^97^
Fibroblasts	Human	Exosomes	Lee *et al*. (2018) ^98^
Urine cells	Human	OCT4, SOX2, KLF4, SV40LT, miR302-367	Wang *et al*. (2013) ^32^
Mesenchymal stem cells	Human	SOX2	Kim *et al*. (2018) ^99^
Adipose-derived stem cells	Human	None (chemical reprogramming)	Park *et al*. (2017) ^66^

**Table 3.** Approaches for neural conversion *in vivo*.

Derived cell type	Starting cell type	Transcription factors used for reprogramming	Reference
Neurons (generic)	Proliferating non-neuronal cells Reactive astrocytes Reactive astrocytes Reactive astrocytes	Ngn2 OligVP16 or Pax6 OligVP16 or Pax6 Ascl1	Grande *et al*. (2013) ^100^ Buffo *et al*. (2005) ^37^ Kronenberg *et al*. (2010) ^38^ Faiz *et al*. (2015) ^101^
	Astrocytes Astrocytes Astrocytes Astrocytes Astrocytes Astrocytes Astrocytes, NG2 cells Reactive astrocytes, NG2 cells Reactive astrocytes, NG2 cells NG2 cells Microglia Neurons (layer IV to V) Neurons (layer II/III to V)	Sox2 Sox2 miR302/367 Ascl1 Sox2 (Niu *et al*. (2013) ^102^) Sox2 and shp53 or shp21 Ascl1, Nurr1, and Lmx1a Neurod1 Ngn2 (+ Bcl2) Sox2 (+ Ascl1) Neurod1 Fezf2 Fezf2	Niu *et al*. (2013) ^102^ Su *et al*. (2014) ^103^ Ghasemi-Kasman *et al*. (2015) ^42^ Liu *et al*. (2015) ^104^ Niu *et al*. (2015) ^105^ Wang *et al*. (2016) ^106^ Torper *et al*. (2015) ^107^ Guo *et al*. (2014) ^108^ Gascon *et al*. (2016) ^83^ Heinrich *et al*. (2014) ^109^ Matsuda *et al*. (2019) ^35^ De la Rossa *et al*. (2013) ^110^ Rouaux and Arlotta (2013) ^111^
Dopaminergic neurons	Reactive astrocytes	Ascl1, Neurod1, Lmx1a, and miR218	Rivetti di Val Cervo *et al*. (2017) ^84^
Interneurons	NG2 cells	Ascl1, Nurr1, and Lmx1a	Pereira *et al*. (2017) ^112^

Since the beginning of this century, the cell programming toolbox has expanded rapidly. The aims of this review are to concisely recapitulate recent advances in this field, to briefly sum up our current understanding of general mechanisms underlying cell fate conversion, to summarize commonalities and differences between the available methods, and to discuss their pros and cons with respect to biomedical applications such as disease modeling and neuroregeneration.

## Boosting transdifferentiation efficiency and fine-tuning sublineage specification

The first seminal reports on transdifferentiating somatic cells into neurons raised strong interest to make this process more efficient and, in particular, to tailor it toward the generation of distinct neural subpopulations. Since neurons are generally post-mitotic, conversion efficiency is a major limiting factor. Several studies addressed this bottleneck. It has been shown that modulation of signaling pathways by small molecules significantly improves iN conversion. For example, combined inhibition of SMAD and GSK3 signaling in human fibroblasts by small molecules can increase iN purity and yield (percentage of neurons in relation to the initial number of plated cells) to up to 80% and 210%, respectively ^52^. Following up on this observation, Pfisterer *et al*. screened five annotated compound libraries for small molecules positively affecting fibroblast-to-neuron transdifferentiation and identified additional pathways (for example, cAMP signaling), whose modulation can increase neuronal yield ^58^. More recently, Herdy *et al*. reported that combining JAK2 inhibition (promoting cell cycle arrest and mesenchymal-to-epithelial transition) with integrin and RAF1 activation (facilitating morphological rearrangements) as well as HIF1α inhibition (fostering the switch from glycolysis to oxidative phosphorylation) efficiently improves human fibroblast-to-neuron conversion ^62^. Moreover, in inducible viral systems, delivering multiple programming factors by all-in-one, polycistronic vectors ^62^ and including a recovery phase between viral transduction and transgene activation ^54^ have been shown to increase conversion efficiency. Lastly, reducing reprogramming barriers in somatic cells, such as inhibiting REST signaling in human fibroblasts ^60^, overcoming senescence ^55^, or inducing epigenetic remodeling by *TET1* activation ^70^, has been reported to boost iN generation, too.

In addition to increasing iN conversion efficiency as such, the generation of defined neuronal subpopulations has been a key focus of this emerging field. While the initial TF combinations used for iN generation resulted primarily in excitatory neurons, these cultures also contained inhibitory GABAergic neurons ^18, 19, 35, 47, 52^. However, some groups reported on iN paradigms that strongly enrich for either glutamatergic ^63– 65^ or GABAergic ^33, 34, 66, 67^ neurons. With respect to potential clinical prospects, the controlled induction of midbrain dopamine neurons and striatal medium spiny neurons (MSNs)—the prime targets of Parkinson’s disease (PD) and Huntington’s disease (HD), respectively—remains a key focus. In order to derive dopaminergic iNs from human fibroblasts, the classic iN reprogramming cocktail of ASCL1, BRN2, and MYT1L can be combined with the dopaminergic fate-specifying TFs LMX1A and FOXA2 ^17^ or a further enriched combination of LMX1A, LMX1B, FOXA2, and OTX2 ^50, 54^. Alternatively, Ascl1 alone has been shown to be sufficient to induce a dopaminergic fate in fibroblasts when combined with Nurr1 and Lmx1a ^69^; Nurr1, Lmx1a, Foxa2, Pitx3, and En1 ^68^; or Nurr1, Lmx1a, and miR124 ^70^. For the derivation of MSNs, combined overexpression of the CNS-enriched miR9/9* and miR124 with MYT1L and the striatal TFs CTIP2, DLX1, and DLX2 was used to convert human fibroblasts into mainly DARPP32-positive GABAergic neurons ^71^. Direct conversion has also been used to generate serotonergic neurons ^73, 74^ as well as peripheral sensory neurons ^80, 81^ and motor neurons (MNs) ^75– 78^ (see Table 1 for further details). A number of these directly converted neuronal subpopulations have been successfully used for *in vitro* disease modeling ^72, 77, 81^ and drug testing ^77^ (for the use of iNs in disease modeling, see also Drouin-Ouellet *et al*. ^113^). In parallel to improving trans-germ layer conversion, the generation of neuronal subtypes from astrocytes has been refined. Pioneering studies by Berninger and Götz already indicated that overexpression of Ngn2 yields mostly glutamatergic neurons whilst direct conversion of astrocytes with Dlx2 results in neurons biased toward a GABAergic phenotype ^82, 114^. More recently, *in vivo* transdifferentiation into neurons with a predominantly dopaminergic fate was achieved ^84^.

With regards to fine-tuning direct conversion paradigms, it is worth mentioning that experimental tools other than classic retroviral or lentiviral systems have been employed for the delivery of TFs or activation of endogenous reprogramming-inducing genes. These include non-integrating viruses ^30, 46, 53^, plasmids and episomal vectors ^45, 85, 86, 92, 94, 96^, pro-neural exosomes released upon ultrasound stimulation ^98^, mRNAs ^97, 99^ and microRNAs ^51^, proteins ^93, 95^, or even transdifferentiation paradigms based solely on chemical cocktails ^47, 64, 66, 87– 91^.

## Forward programming as fallout of transcription factor-based somatic cell fate conversion

Given the tremendous efficacy of TFs in converting somatic cell fates, it is not surprising that this concept has been rapidly adopted to instruct cell fates from pluripotent stem cells (PSCs), thereby replacing or supplementing classic differentiation paradigms using extrinsic factors. “Forward programming” approaches such as the overexpression of neurogenins (NGNs) ^115– 120^ or ASCL1 ^63, 121^ in human PSCs significantly accelerate neuronal differentiation and maturation times. These PSC-derived human neurons have been shown to become electrophysiologically functional as early as two weeks after *NGN* induction ^115, 116^, and synchronized network activity can be detected already after three weeks in culture ^122^. This acceleration is associated with an increased synchronization of the differentiation process, which facilitates disease modeling applications focusing on functional phenotypes as, for example, in schizophrenia and autism ^123, 124^ and tuberous sclerosis and epilepsy ^115, 117^. As with somatic cell fate conversion, combined overexpression of classic neurogenic TFs with TFs promoting distinct regional fates can be used to further fine-tune the generation of distinct neuronal subtypes. For example, overexpression of ASCL1 along with the midbrain-associated TFs NURR1 and LMX1A in human iPSCs has been demonstrated to yield neuronal cultures enriched for TH-positive dopamine-like neurons ^125^. The TFs Ngn2 and Isl1 in combination with Lhx3 and Phoxa2 instruct mouse ESCs to differentiate into cholinergic spinal and cranial MNs, respectively ^126^.

## A need for pioneers?

Pioneer TFs are defined as TFs being able to bind to and open up closed chromatin. Therefore, pioneer TFs can not only induce their own target genes in non-permissive epigenetic states but also enable binding and regulation of secondary TFs. By this mechanism, pioneers are thought to specifically orchestrate the acquisition of new cell fates. Dissecting the process of iN reprogramming, Wapinski *et al*. ^127^ demonstrated in 2013 that Ascl1 acts as a neuronal pioneer TF exactly in this manner: Ascl1 binds almost the same target genes in NSCs and fibroblasts, although these sites are mostly in closed chromatin states in fibroblasts. In contrast to Ascl1, Brn2 and Myt1l preferentially bind to open and accessible chromatin regions. In the context of iN reprogramming with the Ascl1, Brn2, and Myt1l cocktail, Ascl1 at least partially mediates the recruitment of Brn2 and thereby regulates the binding of Brn2 to a proportion of its pro-neural target genes ^127^. Moreover, Ascl1 alone is, in principle, sufficient to induce a neuronal state in fibroblasts, although transdifferentiation with Ascl1 in conjunction with Brn2 and Myt1l is far more efficient and exhibits faster maturation dynamics ^63^.

It is worth mentioning that overexpression of different neuronal pioneer TFs in otherwise identical cellular contexts might lead to varying results. This was recently exemplified by Aydin *et al*., who overexpressed Ascl1 or Ngn2 in isogenic mouse ESC lines ^128^. The authors report that although Ascl1 and Ngn2 did not differ in their capacity to target inaccessible (and accessible) genomic regions, their individual binding patterns are largely non-overlapping. In fact, 90% of all targeted sites were found to be differentially bound by the two TFs as a consequence of their bHLH domain-mediated specificity to distinct E-box motifs. As Ascl1 and Ngn2 both increase chromatin accessibility at their respective target sites, they recruit shared downstream TFs such as Brn2 to different genomic sites, thereby leading to distinct patterns of transcriptional activity. Thus, albeit equivalently inducing pan-neuronal genes, the divergent binding of Ascl1 and Ngn2 elicits distinct neuronal subtype-specific signatures ^128^.

Notably, the effect of a given pioneer TF in PSCs might be quite different from that in somatic cells. This was demonstrated in 2016 by Smith *et al*., who studied the effect of NGN2 overexpression in human fetal fibroblasts ^59^. They revealed that although NGN2 is able to act as a pioneer TF in this transdifferentiation setting (that is, targeting regions in a closed chromatin state), converting fibroblasts into iNs with NGN2 alone is extremely inefficient. However, this low efficiency is significantly enhanced by the small molecules forskolin and dorsomorphin, which promote chromatin accessibility at pro-neural NGN2 binding sites. More specifically, forskolin and dorsomorphin enhance the enrichment of CREB1 at sites bound by NGN2, thereby inducing the expression of the pro-neural gene *SOX4*. SOX4, in turn, elicits further downstream chromatin remodeling and consequently facilitates the activation of other pro-neural genes such as *NEUROD1* and *NEUROD4* ^59^. For other somatic cell types, different pioneer factors might be required to promote cell fate conversion. In mouse microglia, for instance, not Ngn2 or Ascl1 but Neurod1 acts as a neuronal pioneer TF, specifically inducing transcription of its bivalently marked pro-neural target genes ^35^. Along the same lines, oligodendrocytes, which also feature bivalent histone modifications at pro-neural Neurod1 target genes, were successfully reprogrammed into neurons by Neurod1 overexpression ^35^.

As the reprogramming field progressed, major advances were made in profiling cell fate trajectories by single-cell RNA sequencing (scRNAseq). Using this technology, Treutlein *et al*. studied the conversion of mouse embryonic fibroblasts to iNs and specifically analyzed the contribution of the neuronal pioneer TF Ascl1 to the induction and stabilization of the fibroblast-to-iN fate switch in the Ascl1–Brn2–Myt1l paradigm ^129^. Concordant with the results of Wernig’s group, they showed that overexpression of Ascl1 alone is sufficient to homogenously induce downregulation of fibroblast-enriched transcriptomic signatures, to upregulate the expression of neuronal genes, and to promote cell cycle exit. They found the continued expression of Ascl1 as well as co-expression of Brn2 and Myt1l to be essential for the stabilization of neuronal fate and subsequent neuronal maturation, whereas silencing of Ascl1 in the course of the conversion process resulted in the reappearance of fibroblast signatures ^129^. Notably, the majority of Ascl1-only-induced cells do not acquire a neuronal identity, even if Ascl1 expression levels are maintained, but activate a myocyte-related transcriptional program ^129^. This observation might be explained by the lack of *Myt1l* induction in Ascl1-only conditions. Mall *et al*. investigated the role of this non-pioneer TF during fibroblast-to-neuron conversion and revealed that its main function is to interact with the Sin3b–HDAC1 complex to repress non-neuronal transcriptional programs ^130^. Myt1l-repressed targets include genes promoting proliferation, such as *Hes1*, and genes inducing alternative lineages, including targets relevant for myocyte differentiation ^130^. Together, these data indicate that, in addition to pioneer factors, secondary fate-specifying or alternative fate-repressing cues (or both) are necessary to ensure proper phenotype stabilization. Consequently, Tsunemoto *et al*. recently screened a library of 598 TF pairs for their ability to convert mouse embryonic fibroblasts into functional iNs ^49^. As expected, almost all successful combinations included at least one member of the Ascl, Ngn, or Neurod families. However, pairs of pro-neural TFs comprising no pioneer TF also yielded functional iNs, demonstrating that pioneer TFs are not an indispensable condition for direct cell fate conversion ^49^. Along similar lines, the group of Lei Qi performed a CRISPR activation screen to identify single TFs and TF combinations that promote differentiation of mouse ESCs and direct conversion of fibroblasts into neurons ^48^. In addition to known pro-neural TFs such as Ngns or Brn2, their top hits included non-pioneer TFs and even non-neural-specific TFs such as the epigenetic regulator Ezh2 ^48^.

Taken together, the currently available data support a two-stage architecture of the conversion process. First, target cell type-specific genes need to be made accessible in case they are in an unfavorable chromatin state in the starting cell type. In addition to pioneer TFs, epigenetic modifiers or other factors modulating chromatin accessibility can exert this effect. Overexpression of miR9/9* and miR124, for instance, has been shown to promote gradual remodeling of chromatin accessibility at fibroblast-specific enhancers (change to closed chromatin) and chromatin opening at pan-neuronal gene loci ^78^. Second, after induction of epigenetic plasticity, acquisition and stabilization of a new cell fate have to take place. Although this process can be initiated and orchestrated by pioneer TFs too, it mostly involves additional TFs. These can be co-transduced in the starting cell along with the pioneer TF (that is, by overexpressing TF combinations) or induced by small molecules used to promote the direct conversion process or they are direct transcriptional targets of the pioneer TF and thus secondarily induced by the pioneer itself. Eventually, pioneer as well as non-pioneer TFs instruct the adoption of a specific cell fate through either active induction of target lineage-specific genes (as was demonstrated for, for example, Ascl1 ^129^ or Neurod1 ^35^) or transcriptional repression of genes instructing alternative cell fates (as shown for, for example, Myt1l ^130^). Notably, however, the process of fate acquisition might involve additional intermediate steps, since scRNAseq time-course analyses of the iN conversion process indicate the presence of transient, unstable progenitor-like identities before a stable neuronal phenotype is adopted ^34, 129^. As overarching mechanistic principles underlying cell fate conversion become increasingly uncovered, it is important to note that the exact mechanisms of fate switches will always comprise components highly specific to the identity of the interconverted cell types and the individual conversion paradigm.

## Tampering with epigenetic age

The epigenetic memory of a cell falls into two major categories: cell fate and age. Since significant transcriptomic and epigenetic remodeling plays a pivotal role in the process of cell programming, it seems natural to ask how different programming paradigms affect a cell’s aging signature. However, age is a highly multi-faceted phenomenon and hard to assess by simple means (see the 2015 review by Studer, Vera, and Cornacchia ^131^). Some aspects of cellular aging, such as compromised nuclear architecture, cannot be easily assessed in a quantitative manner. Others, such as telomere length, might not strictly correlate with biological age, depending on the tissue context ^132^. One alternative way to estimate the biological age of a cell independent of its somatic cell fate is to analyze DNA methylation (DNAm) signatures and apply algorithms calculating a DNAm age ^133^. When applied to iPSC generation, DNAm ages have been shown to be reset upon induction of pluripotency, which is in line with the fact that iPSC reprogramming resets the starting cell’s identity back to an embryonic-like state ^133, 134^. Thus, somatic-to-iPSC reprogramming represents a tool to derive epigenetically rejuvenated cells. Conversely, in 2015, the groups of Yixuan Wang and Fred Gage demonstrated that aging hallmarks such as age-specific transcriptional signatures and the age-dependent loss of nucleocytoplasmic compartmentalization are preserved in mouse ^135^ and human ^56^ iNs, respectively. One year after these reports, it was demonstrated that the DNAm ages of iNs are retained, too, and almost perfectly correlate with their donors’ chronological ages ^57^. Over the last two years, several other studies corroborated the notion that age-associated cellular alterations such as senescence, susceptibility to DNA damage, mitochondrial defects, loss of heterochromatin, and alterations in nuclear organization are preserved in fibroblast-derived iNs ^61, 79^. These findings indicate that iNs maintain not only epigenetic but also functional age-related phenotypes of their cells of origin. Interestingly, all of these studies were conducted in a scenario where the converted cells immediately enter the post-mitotic stage of an iN. Thus, we became interested in the question of how age preservation would work upon transdifferentiation into a proliferative somatic stem cell population. Following up on this idea, we used temporary overexpression of SOX2 and c-MYC to convert adult peripheral blood cells into induced NSCs (iNSCs). Using the Horvath and other epigenetic age predictor algorithms, we found that iNSCs generated in this manner undergo massive epigenetic rejuvenation similar to what is observed during iPSC reprogramming ^30^. This observation is noteworthy, as our conversion approach is OCT4-free and there is no evidence of a transit through a pluripotent state. Although the mechanism underlying the reset of biological age remains to be unveiled, this finding strongly suggests that epigenetic rejuvenation does not require an intermediate pluripotent stage and can also be achieved during transdifferentiation of somatic cell types. This notion is further supported by very recent analyses of DNAm changes upon iPSC reprogramming, which show that the reset of DNAm age and the establishment of a stable and self-sustaining pluripotent state follow different time dynamics ^136^. From a conceptual point of view, these observations support the idea that epigenetic rejuvenation, in principle, can be achieved in somatic cells.

The question of whether or not reprogrammed cells preserve age signatures is especially relevant when it comes to modeling age-related diseases. In particular, successful modeling of neurodegenerative diseases might depend on the preservation of cellular defects naturally accumulating over an organism’s life span. The importance of age preservation for disease modeling was recently illustrated in the context of HD. The group of Andrew Yoo found that aggregation of the disease-causing mutant huntingtin protein can be readily recapitulated in directly converted MSNs but not in iPSC-derived MSNs, a phenomenon the authors attributed to the erasure of age signatures such as the restoration of proteasomal activity in iPSC-derived MSNs ^72^. Acknowledging that the lack of aging hallmarks in iPSC-derived somatic cells can impede modeling of age-associated pathophenotypes, strategies such as progerin overexpression or telomerase inhibition have been explored to promote the emergence of age-associated phenotypes ^137, 138^. Owing to their age memory, directly converted neurons might not require additional age-promoting treatments for modeling late-onset neurodegenerative diseases, for example. However, it is fair to say that iNs might, vice versa, be less suitable for modeling neurodevelopmental disorders.

## Somatic memory and authenticity

Although some diseases affect neurons rather broadly, others are known to target preferentially specific subtypes such as PD, which is associated with a loss of mesencephalic dopaminergic neurons in the substantia nigra. Since cellular pathomechanisms might be cell type dependent, the authenticity of the transdifferentiated neural subpopulation might contribute significantly to the validity and power of cellular disease models. While there is evidence for low levels of residual somatic memory in low-passage iPSCs ^134, 139, 140^, these signatures appear to vanish after prolonged *in vitro* cultivation ^140^. This presents differently in directly converted cells. Tsunemoto *et al*. analyzed four fibroblast-derived iN populations reprogrammed by different TF combinations and revealed that although the global transcriptome of iNs is highly similar to that of endogenous neurons, all iN populations showed residual low-level expression of a subset of fibroblast-specific genes ^49^. Residual somatic signatures were also recently reported for iNSCs. Thier *et al*. derived iNSCs with neural plate border identity from different populations of human fibroblasts and blood cells ^31^. They found that dermal fibroblast-derived but not blood-derived iNSCs still express the fibroblast marker *COL3A1*, although other fibroblast-lineage markers are significantly downregulated upon transdifferentiation ^31^. Nevertheless, residual somatic signatures in directly converted cells appear to be insufficient to maintain the identity and function of the cell of origin. For example, hepatocyte-derived iNs were shown to retain more than 10% of the liver-specific transcriptomic signature, but resulting iNs are capable of neither secreting albumin nor producing urea ^44^.

However, the questions of whether and to what extent the function of the converted iNs can be compromised by residual somatic signatures of the donor cell certainly merit further investigation, and recent data suggest that authenticity is an issue not restricted to direct cell fate conversion. Ichida *et al*. compared primary mouse spinal MNs with ESC-derived, iPSC-derived, and directly converted MNs and revealed that all *in vitro*-derived MN populations, regardless of the reprogramming paradigm used, expressed only about 55% to 86% of the primary MN transcriptome ^75^. These differences were accompanied by even more pronounced discrepancies in the methylation status ^75^. From a technical point of view, this study illustrates a fundamental bottleneck of contemporary cell fate reprogramming and conversion research: traditionally, cell fate identification has been based mostly on the expression of cell type-specific marker profiles, and, if applicable, further characteristic features such as specific functional properties, including electrophysiological data, have been considered. These approaches are biased, however, as they are hypothesis driven. More holistic approaches such as the in-depth analysis of transcriptomic data, ideally in single-cell resolution, and comparative methylation analysis, as performed by the Eggan lab, might represent means to provide more reliable and biologically meaningful measures of cell identity and authenticity. From a biological perspective, such findings also point to more general limitations of *in vitro* cell systems in recapitulating *in vivo* scenarios. On the other hand, it remains unclear what degree of somatic authenticity is eventually required to, for example, recapitulate disease-specific phenotypes—an issue which also depends on the specific experimental hypothesis. For other biomedical applications such as replacement of distinct neuronal subpopulations, utmost authenticity will always represent the ultimate goal.

## Destabilizing and converting cell fates *in vivo*

Translation of *in vitro* paradigms of direct cell fate conversion to an *in vivo* scenario remains one of the most fascinating perspectives of regeneration. From a translational point of view, such approaches could eventually replace cell transplantation. From a biological perspective, transdifferentiation of region-specific cells in a native tissue environment might represent the ultimate approach to approximate authenticity.

In the CNS, the longest history of *in situ* transdifferentiation has astrocyte-to-neuron conversion ( Table 3), starting with the observation that antagonizing Olig2 or overexpressing Pax6 after traumatic brain injury enables neurogenesis from resident reactive astrocytes ^37^; this phenomenon has also been recapitulated after infliction of mild brain ischemia ^38^. Similarly, Neurod1 has been shown to convert reactive astrocytes as well as NG2-positive progenitors into neurons in mouse stab injury and Alzheimer’s disease models ^108^. Heinrich *et al*. demonstrated that retrovirus-mediated overexpression of Sox2 alone or in combination with Ascl1 transdifferentiates NG2 cells into neurons in the acutely injured cortex ^109^. Astrocyte-to-neuron conversion has also been achieved in the healthy, unlesioned rodent CNS, for example, by the overexpression of the pioneer TFs Ascl1 ^101, 104^ and Sox2 ^102, 103, 106^ or mediated by miR302/367 ^42^.

As with *in vitro* conversion, *in vivo* transdifferentiation is being increasingly refined toward the generation of distinct neuronal subpopulations. Torper *et al*. ^107^ tested the TF combination of Ascl1, Nurr1, and Lmx1a, which specifies dopaminergic-like neurons from human PSCs ^125^ and fibroblasts ^69^ *in vitro*. Notably, although this TF cocktail successfully converted astrocytes and NG2 glia into neurons *in vivo*, these neurons did not adopt a dopaminergic phenotype ^107^. Instead, this TF combination was found to promote the generation of interneurons exhibiting a fast-spiking parvalbumin-positive phenotype ^107, 112^, highlighting the necessity to re-assess tools developed *in vitro* for their applicability *in vivo*. The team of Ernest Arenas then showed that supplementation of the TF combination of Ascl1 and Lmx1a with Neurod1 and miR218 can successfully instruct the conversion of astrocytes to dopamine neurons, which alleviated gait impairments in a mouse model of PD ^84^, emphasizing the relevance of this approach for clinical translation. Recent work by Matsuda *et al*. extended *in vivo* transdifferentiation to mouse microglia, which they converted with Neurod1 into striatal projection neuron-like cells, which were electrophysiologically active and formed excitatory synapses with host neurons ^35^. Interestingly, even post-mitotic neurons appear to be amenable to TF-based fate shifting: the TF Fezf2 was shown to be competent of re-specifying post-mitotic mouse layer II/III callosal projection ^111^ and layer IV spiny neurons ^110^ into layer V corticofugal projection neurons.

Escalating the concept of TF-mediated *in vivo* cell fate shifts, the results of several studies point to the possibility of reprogramming cells *in situ* toward pluripotency ^141– 144^. Interestingly, in accordance with the concept of partial reprogramming, the group of Juan Carlos Belmonte demonstrated that short-term cyclic expression of Oct3/4, Sox2, Klf4, and c-Myc *in vivo* does not lead to the establishment of a stable pluripotent fate but can increase the regenerative capacity of multiple organs in physiologically aged mice and promote cellular rejuvenation in progeria mice suffering from premature aging ^145^.

## Challenges for clinical application

Even though *in vivo* conversion is a promising strategy to exploit endogenous sources for cell replacement, a number of limitations have to be overcome before this approach is fit for clinical translation. First, the delivery of fate-instructing factors has to be good manufacturing practice compliant and applicable in living humans. Here, established viral vector systems successfully applied in gene therapy approaches such as adeno-associated viruses ^104, 107, 112^ might represent an attractive solution. Alternatively, non-viral approaches such as transducible proteins, mRNAs, or small molecules might qualify for delivering the required cell programming cues *in vivo* (for a review on recent technologies facilitating *in vivo* reprogramming, see Larouche and Aguilar ^146^). Whatever delivery system is chosen, it has to enable factor distribution to the lesion site. For focal lesions, stereotaxic delivery can be considered, but more global cell loss might require modes of systemic administration that are not impeded by the blood–brain barrier. Moreover, dependent on the individual transdifferentiation regimen, multiple rounds of factor administration or delivery of depots such as scaffold-bound conversion factors might be necessary (reviewed in 2019 by Larouche and Aguilar ^146^ and Bruggeman *et al*. ^147^).

*In vivo* conversion is further complicated by the fact that this process can be strongly context dependent. Grande *et al*. showed that Ngn2-mediated conversion of proliferating non-neuronal cells yields GABAergic neurons in the mouse striatum but results in the emergence of glutamatergic neurons in the neocortex ^100^. In addition, the local microenvironment can affect conversion efficiency. Wang *et al*. ^106^ found that decreasing p53–p21 signaling increases the yield of astrocyte-derived neuroblasts by preventing p53-induced cell cycle exit, while locally secreted neurotrophins can support their maturation ^105^. Götz and her team reported that counteracting oxidative stress and ferroptosis can significantly increase neuron derivation from glial cells *in vivo* ^83^.

Another risk factor to be considered in the context of *in vivo* conversion is the emergence of partially programmed cells, the potential tumorigenicity of such cells, and their potential impact on tissue homeostasis. Finally, as *in vivo* conversion efficiencies increase, depletion of the target cell population can become a serious issue. This is particularly true for astrocytes, oligodendrocytes, and microglia, which serve a plethora of vital functions in tissue homeostasis and neuronal function. In this context, cells with residual self-renewal capacity might serve as particularly attractive targets for neuronal conversion.

## Implications

**Figure 1.**
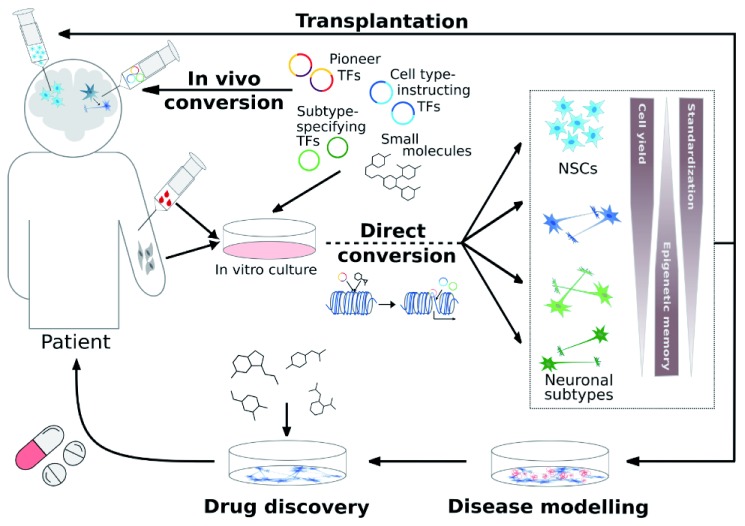
Direct cell fate conversion strategies in the context of biomedical applications. Depending on the choice of programming factors, direct conversion can be fine-tuned to derive different cell types and even distinct neuronal subtypes, which can serve as platforms for disease modeling and drug discovery or as donor source for neural transplantation. Notably, different cell fate programming paradigms are characterized by varying degrees of scalability (that is, cell yield), retention of epigenetic memory, and standardization (for example, cell culture homogeneity and feasibility to provide quality-controlled batches), which might influence their applicability for biomedical applications. In contrast to transplantation of *in vitro*-derived cells, *in vivo* cell fate conversion might enable restoration of neuronal circuitry from endogenous sources. NSC, neural stem cell; TF, transcription factor.

Notwithstanding the many fundamental and translational questions that remain to be addressed in the context of direct cell fate conversion, this field provides fascinating prospects for a number of biomedical applications ranging from disease modeling via drug discovery to cell therapy and endogenous regeneration ( Figure 1). For disease-related research, the prospect of age preservation in iNs could render these cells a preferred resource for patient-specific modeling of late-onset neurodegenerative disorders and establishing *in vitro* systems for compound screening. As for regeneration, somatic cell fate conversion and TF-based forward programming of PSCs could enable intricate approaches for generating neural subtypes faster and with much higher precision than conventional methods. Finally, *in vivo* transdifferentiation is about to revolutionize our concepts for neuroregeneration and, for some applications, might eventually substitute traditional cell transplantation strategies. However, although epigenetic remodeling is a general principle underlying cell programming, the preservation of somatic and age memory seems to be unique for each conversion paradigm. Thus, developing a better understanding of the mechanisms underlying specific conversion trajectories is essential in order to fully exploit this emerging technology for biomedical research and therapy.

## Abbreviations

Ascl1, Achaete-scute homolog 1; bHLH, basic helix–loop–helix; Brn2 aka POU3F2, POU domain class 3 transcription factor 2; Brn4 aka POU3F4, POU domain class 3 transcription factor 4; cAMP, cyclic adenosine monophosphate; CNS, central nervous system; c-Myc, avian myelocytomatosis viral oncogene cellular homolog; COL3A1, collagen type III alpha 1 chain; CREB1, cAMP responsive element-binding protein 1; CRISPR, clustered regularly interspaced short palindromic repeats; CTIP2 aka BCL11B, B-cell lymphoma/leukemia 11B; DARPP32 aka PPR1B, protein phosphatase 1 regulatory subunit 1B; Dlx, distal-less homeobox; DNAm, DNA methylation; EGF, epidermal growth factor; En, engrailed homeobox; ESC, embryonic stem cell; Ezh2, enhancer of zeste homolog 2; Fezf2, Fez family zinc finger protein 2; FGF, fibroblast growth factor; Fox, forkhead box; GABA, gamma aminobutyric acid; GSK, glycogen synthase kinase; HD, Huntington’s disease; HDAC1, histone deacetylase 1; Hes1, hairy and enhancer of split-1; HIF1α, hypoxia-inducible factor 1-alpha; iN, induced neuron; iNSC, induced neural stem cell; iPSC, induced pluripotent stem cell; ISL1, islet1; JAK2, Janus kinase 2; Klf4, Krueppel-like factor 4; LIF, leukemia inhibitory factor; LHX3; LIM homeobox 3; Lmx1, LIM homeobox TF 1; MN, motor neuron; MSN, medium spiny neuron; Myod3, myogenic differentiation 3; Myt1l, myelin transcription factor 1 like; Neurod1, neurogenic differentiation 1; NG2, neuron-glial antigen 2; NGN, neurogenin; NPC, neural progenitor cell; NSC, neural stem cell; NURR1, nuclear receptor related 1; Oct3/4, octamere-binding transcription factor 3/4; Olig, oligodendrocytes TF; OTX, orthodenticle homeobox; PAX, paired box protein; PD, Parkinson’s disease; PHOX2A, paired like homeobox 2A; PSC, pluripotent stem cell; Pitx, pituitary homeobox; RAF1, rapidly accelerated (rat) fibrosarcoma 1; REST, RE1 silencing TF; scRNAseq, single-cell RNA sequencing; Sin3b, SWI-independent transcription regulator family member b; Sox2/SOX4, sex determining region Y-box 2/4; TET, Ten-eleven translocation methylcytosine dioxygenase; TF, transcription factor; TGF, transforming growth factor; TH, tyrosine hydroxylase
